# Emotional regulation mechanisms regarding posttraumatic and depressive symptoms in maltreated youth

**DOI:** 10.3389/fpubh.2025.1527120

**Published:** 2025-02-27

**Authors:** Kinsey Ellis, Christopher A. Kearney

**Affiliations:** Department of Psychology, University of Nevada, Las Vegas, Las Vegas, NV, United States

**Keywords:** maltreatment, emotional regulation, expressive suppression, cognitive reappraisal, posttraumatic stress, depression

## Abstract

Maltreated youth are a particularly vulnerable group that often contends with multifaceted mental health challenges, especially symptoms of posttraumatic stress and depression. Emotional dysregulation may have a central role in linking victimization to youth psychopathology in this population, but few have examined the effect of specific mechanisms such as expressive suppression and cognitive reappraisal in this regard. The present study examined these mechanisms vis-à-vis posttraumatic and depressive symptoms in a highly diverse sample of 133 youth aged 11–17 years who were removed from home following maltreatment. Expected positive relationships were found with respect to expressive suppression and total and cluster (i.e., intrusion, avoidance, alterations in cognition and mood, and alterations in arousal and reactivity) posttraumatic stress symptoms as well as depressive symptoms. Conversely, unexpected positive relationships were found with respect to cognitive reappraisal and these symptoms only for specific groups, notably males, younger youth, and those who had not experienced sexual maltreatment. The findings may have implications for tailoring clinical protocols for this vulnerable population, and may offer directions for integrating expressive suppression and cognitive reappraisal into transdiagnostic models of psychopathology in youth.

## 1 Introduction

A particularly vulnerable pediatric group that is of substantial concern to mental health professionals is youth who have experienced maltreatment and who have been removed from home (1). These individuals often endure multiple traumatic events in addition to the stressors of foster care placement, school instability, and loss of social support networks (2). As such, maltreated youth are at substantial risk for multifaceted psychiatric problems that most commonly include symptoms of posttraumatic stress disorder and depression (3). These symptoms can impair cognitive and social functioning and exacerbate anger, anxiety, and somatic complaints, among other problems [e.g., (4)]. Minoritized maltreated youth, and in particular multiracial maltreated youth, are at increased risk for these mental health challenges, thus requiring diverse research samples (5).

Investigations of primary connections between child maltreatment and posttraumatic and depressive symptoms have centered on the key role of emotional dysregulation, a transdiagnostic construct involving difficulty recognizing, evaluating, modifying, and managing emotions in personal and socially acceptable ways (6). Some deficits related to emotional dysregulation apply more generally to posttraumatic and depressive challenges, such as excessive reactivity to stimuli, problems in threat and reward processing, and interference in learning and goal attainment (7). Other deficits related to emotional dysregulation apply more specifically to posttraumatic and depressive challenges, such as those involving under-regulation (e.g., distress, irritability/anger, hypervigilance) or over-regulation (e.g., thought suppression, avoidance) (8).

Research with respect to the specific mechanisms surrounding the role of emotional dysregulation in posttraumatic and depressive symptoms, and particularly among a highly vulnerable group such as maltreated youth, remains only emergent. Two mechanisms that may help explain this relationship include expressive suppression and cognitive reappraisal. Expressive suppression involves inhibiting the outward expression of emotion; cognitive reappraisal involves reframing an emotion-eliciting situation to modify its impact (9). Expressive suppression is associated with reduced positive affect and increased negative affect, whereas cognitive reappraisal is associated with greater experience and expression of positive emotions (10, 11). Expressive suppression is thus considered to be a more maladaptive coping strategy, whereas cognitive appraisal is considered more adaptive (12). Both strategies, however, may be influenced by contexts such as type of trauma, attachment, cultural identity, and developmental status, among other factors [e.g., (13)]. Although expressive suppression and cognitive reappraisal have been linked to posttraumatic and depressive symptoms in adults, little is known with respect to these mechanisms in maltreated youth.

The present study examined expressive suppression and cognitive reappraisal among a highly diverse set of youth that had experienced traumatic maltreatment events sufficient enough to warrant removal from home. Expressive suppression was expected to positively predict total and cluster (i.e., intrusion, avoidance, alterations in cognition and mood, and alterations in arousal and reactivity) symptoms of posttraumatic stress as well as depressive symptoms. Conversely, cognitive reappraisal was expected to negatively predict each of these symptom groups.

## 2 Materials and methods

### 2.1 Participants

Participants were 133 youth aged 11–17 (M = 14.08, SD = 1.81) years in Department of Family Services (DFS) facilities within a large urban area. Participants were male (47.4%), female (46.6%), transgender (3.1%), and nonbinary (3.0%). Participants self-identified as African American/Black (35.5%), Multiracial (23.3%), White (18.8%), Hispanic/Latinx (12.0%), Asian (2.3%), Other (7.5%), and Native American (0.8%). Participants were youths in DFS custody referred for psychological evaluation following removal from their primary caregiver for reasons including psychological maltreatment (59.4%), neglect (58.6%), physical maltreatment (48.1%), and/or sexual maltreatment (25.6%). Evaluations typically occurred between 1 month and 1 year following residential removal.

### 2.2 Measures

#### 2.2.1 UCLA Post-Traumatic Stress Disorder Reaction Index

The UCLA PTSD-RI is a semi-structured interview for trauma exposure and reactions in youth aged 7–18 years (14). The measure assesses PTSD symptom clusters (intrusions, avoidance, negative alterations in mood and cognition, negative alterations in arousal and reactivity), frequency of PTSD symptoms, and distress and impairment across settings. The measure has good internal consistency (*α* = 0.88–0.94), factor structure, discriminant validity, and diagnostic clarification accuracy in maltreated youth (15, 16). Symptom cluster scores were evaluated for the present study. Cronbach alphas were calculated for the present sample for total score (*α* = 0.94), intrusions (*α* = 0.85), avoidance (*α* = 0.60), negative alterations in cognitions and mood (*α* = 0.88), and negative alterations in arousal and reactivity (*α* = 0.78).

#### 2.2.2 Revised Children’s Anxiety and Depression Scale

The RCADS is a 47-item measure of generalized anxiety, separation anxiety, social phobia, panic, and obsessive-compulsive as well as major depressive symptoms (17). The latter subscale was used in the present study to examine depression symptoms. The RCADS has good internal consistency (*α* = 0.60–0.96) and construct, convergent, and discriminant validity, as does the major depressive symptoms subscale (18, 19). Cronbach’s alpha was calculated for the present sample for the major depression subscale (*α* = 0.89).

#### 2.2.3 Emotion Regulation Questionnaire for Children and Adolescents

The ERQ-CA is a 10-item self-report measure of frequency of emotion regulation strategy utilization in youth aged 9–18 years (20). Four items comprise the cognitive reappraisal (CR) subscale and six items comprise the expressive suppression (ES) subscale. Confirmatory factor analyses have supported the measure’s construct validity [e.g., (21)], and the measure has demonstrated good internal consistency for CR (α = 0.83) and ES (*α* = 0.75) (20). ES and CR item descriptions are paraphrased in the results section. Cronbach alphas were calculated for the present sample for CR (*α* = 0.84) and ES (*α* = 0.62).

### 2.3 Procedure

Procedures were in accordance with university and DFS policies regarding research with human participants, including IRB approval. An interlocal contract was also in accordance with state and county laws regarding the treatment of youth in protective custody. The assessment protocol did not require parental permission given the youths’ DFS status. Eligible youths included those aged 11–17 years at a DFS facility. Youths provided assent, were instructed that they were not obligated to answer any question, and were permitted to withdraw from the study at any time. Assessment procedures were conducted in a confidential environment by a supervised doctoral student or licensed psychologist. Youth were excluded from the study if they did not provide assent, did not endorse experiencing a traumatic event, or did not complete the assessment (*n* = 26). No analyses were conducted on excluded youth. DFS records were utilized to identify maltreatment type.

### 2.4 Data analyses

Linear regression analyses were used to examine expressive suppression (ES) and cognitive reappraisal (CR) ERQ-CA item scores vis-à-vis RCADS total depression, PTSD-RI total symptoms, and PTSD-RI cluster symptom scores. Multicollinearity was assessed by evaluating bivariate correlations between ERQ-CA items and variance inflation factors (VIF) of items in the multiple regression analyses. All bivariate correlations were < 0.70 and all VIF values were < 5; multicollinearity was thus not considered problematic. *Post hoc* regression analyses were conducted to examine the effects of age (younger/older; age 11–15/15–17 years), race/ethnicity, gender, and maltreatment type on the relationship between emotion regulation strategies and outcome variables. *Post hoc* regression analyses were also conducted to examine items and potential interaction effects.

## 3 Results

ES emerged as a significant positive predictor of depression symptoms, total PTSD symptoms, and PTSD symptom cluster symptoms. ES items collectively predicted RCADS total depression scores [adj. *R*^2^ = 22.9%, *F*(4, 120) = 10.23, *p* < 0.001] and PTSD-RI total scores [adj. *R*^2^ = 26.0%, *F*(4, 121) = 11.99, *p* < 0.001]. Items 2 (keep feelings to oneself) and 4 (careful not to show happiness) contributed significantly to both models, and particularly the latter. Items 6 (control feelings) and 9 (careful not to show feeling bad) did not contribute significantly to the models. ES items collectively predicted PTSD-RI cluster scores: intrusion [adj. *R*^2^ = 19.9%, *F*(4, 121) = 8.79, *p* < 0.001], avoidance [adj. *R*^2^ = 8.6%, *F*(4, 121) = 3.94, *p* = 0.005], alterations in cognitions and mood [adj. *R*^2^ = 26.6%, *F*(4, 121) = 12.31, *p* < 0.001], and alterations in arousal and reactivity [adj. *R*^2^ = 20.8%, *F*(4, 121) = 9.19, *p* < 0.001]. Item 4 contributed significantly to each model. Item 2 also contributed significantly to alterations in cognitions and mood and alterations in arousal and reactivity. Items 6 and 9 did not contribute significantly to any of the models. Post-hoc hierarchical regressions were conducted following control of gender, age, race/ethnicity, and type of maltreatment, and findings were confirmed. No significant interaction effects were found.

CR emerged as a significant positive predictor of total PTSD symptoms and some PTSD symptom cluster symptoms, but not of depression symptoms. CR subscale items collectively predicted PTSD-RI total scores [adj. *R*^2^ = 7.3%, *F*(6, 126) = 2.720, *p* = 0.016]. Item 1 (think something different when want to feel happier) contributed significantly to the model. CR items also collectively predicted symptoms of intrusion [adj. *R*^2^ = 9.2%, *F*(6, 126) = 3.234, *p* = 0.005] and alterations in cognition and mood [adj. *R*^2^ = 5.7%, *F*(6, 126) = 2.322, *p* = 0.037], but not of avoidance or alterations in arousal and reactivity. Only Item 1 contributed significantly to both models. Post-hoc analyses were conducted following control of gender, age, race/ethnicity, and type of maltreatment. Findings were confirmed with the exception of Item 1’s contribution to alterations in cognitions and mood. The expected overall inverse relationship was thus not found, but specific interactions are noted next.

Analyses revealed a significant interaction for *gender* [*β* = −0.27, *t*(121) = −2.35, *p* = 0.020] vis-à-vis CR and total PTSD symptoms as well as alterations in cognitions and mood [*β* = −0.18, *t*(121) = −2.18, *p* = 0.031] and alterations in arousal and reactivity [*β* = −0.28, *t*(121) = −2.34, *p* = 0.021]. Simple slope analyses revealed significant positive relationships for males only. Analyses also revealed significant interactions for *maltreatment type*. CR and sexual maltreatment interacted to predict avoidance [*β* = −0.20, *t*(130) = −2.30, *p* = 0.023], alterations in cognitions and mood [*β* = −0.19, *t*(130) = −2.10, *p* = 0.038], and alterations in arousal and reactivity [*β* = −0.23, *t*(130) = −2.52, *p* = 0.013]. CR and neglect interacted to predict avoidance [*β* = −0.31, *t*(130) = −2.30, *p* = 0.023] and alterations in arousal and reactivity [*β* = −0.37, *t*(130) = −2.69, *p* = 0.008]. CR and psychological maltreatment interacted to predict alterations in cognitions and mood [*β* = −0.23, *t*(130) = −2.00, *p* = 0.048] and alterations in arousal and reactivity [*β* = −0.27, *t*(130) = −2.30, *p* = 0.024]. Simple slopes revealed positive relationships between CR and symptoms for youth who had not experienced these maltreatment types, but nonsignificant relationships for youth who had. Analyses also revealed a significant interaction for *age*. CR and age interacted to predict intrusion symptoms [*β* = 0.23, *t*(130) = 2.06, *p* = 0.042]; a positive relationship emerged for youth aged <15 years but not for older youth.

## 4 Discussion

The present study examined potential mechanisms (expressive suppression and cognitive reappraisal) vis-à-vis posttraumatic and depressive symptoms in maltreated youth. As expected, expressive suppression emerged as a significant and positive predictor of total and cluster (i.e., intrusion, avoidance, alterations in cognition and mood, and alterations in arousal and reactivity) symptoms of posttraumatic stress as well as depressive symptoms. Conversely, however, the expected inverse direction with respect to cognitive reappraisal and these symptom groups did not emerge overall. Interaction effects revealed a positive relationship regarding cognitive reappraisal with respect to specific groups, notably males, younger youth, and youth who had not experienced sexual maltreatment, vis-à-vis certain subscales.

Expressive suppression findings were manifested specifically with respect to care about showing happiness. Suppression of positive emotions may thus be a particularly important process regarding posttraumatic and depressive symptoms in maltreated youth. This is consistent with literature that supports links between trauma exposure, difficulty regulating positive emotions, and psychopathology (22). Weiss et al. (23) found that difficulties regulating positive emotions predicted PTSD symptom severity beyond the variance accounted for by difficulties regulating negative emotions in women victimized by interpersonal violence. Links between suppression of positive emotions and depressive symptoms have also been identified (24). Results are also consistent with broader literature proposing that the effects of expressive suppression are influenced by contextual factors such as the valence or type of emotion being suppressed [e.g., (25)].

Cognitive reappraisal findings were more nuanced, with unexpected positive effects noted especially for males, younger youth, and youth who had not experienced sexual maltreatment. Maltreated youth demonstrate trauma-related neural alterations that may impact their ability to utilize cognitive reappraisal effectively [e.g., (26)]. Younger youth and males are particularly less likely to use reappraisal flexibly and effectively (27, 28). Ineffective use of cognitive reappraisal can negatively impact self-efficacy and psychological well-being (29). However, cognitive reappraisal has been linked to resilience and greater adjustment among survivors of sexual maltreatment, which is well-known to be especially pernicious among types of maltreatment (30).

Findings from the present study may thus have implications for clinical practice. With respect to assessment, the nuanced findings reveal the need to delve more deeply into specific aspects of these mechanisms, perhaps via open-ended interviews, responses to hypothetical vignettes, and emotion regulation tasks to reappraise emotional stimuli (31). With respect to treatment, expressive suppression may be an important part of daily mindfulness and cognitive-behavioral practices to enhance emotional regulation (32, 33). The present study also offers insight into possible intervention targets for maltreated youth.

Results from the present study may also be relevant to diagnostic definitions of complex PTSD, particularly with respect to relational trauma and disturbances in self-organization that include affective dysregulation, negative self-concept, and disturbances in relationships (34). In addition, conceptual frameworks for interpreting complex PTSD often focus on how prolonged traumatic, relational stressors disrupt the brain’s predictive processing capabilities, particularly with respect to negative self-concept, biased perceptions toward self-criticism, and social prediction errors that produce mistrust and withdrawal (35). Indeed, impaired cognitive reappraisal with problems in social functioning relate to specific neural circuitry patterns of emotional regulation in patients with PTSD (36).

Limitations of the study should be noted, including reliance solely on youth self-report, lack of knowledge of concurrent trauma events, presence of multiple traumas in some cases, and varied assessment times following removal from home. Despite these limitations, the present study offers potential directions for further research. Examples include greater investigation of expressive suppression and cognitive reappraisal across specific demographic groups, maltreatment types, trauma profiles, and developmental phases. In addition, integrating expressive suppression and cognitive reappraisal into transdiagnostic models of psychopathology in youth would seem desirable. Of special importance in this regard would be utilizing such models to better understand how interventions specifically work to reduce counterproductive, and to enhance productive, emotional regulation strategies, particularly for highly vulnerable populations such as maltreated youth (37).

## Data Availability

The datasets presented in this article are not readily available because data are confidential. Requests to access the datasets should be directed to chris.kearney@unlv.edu.
